# HLA Does Not Impact on Short-Medium-Term Antibody Response to Preventive Anti-SARS-Cov-2 Vaccine

**DOI:** 10.3389/fimmu.2021.734689

**Published:** 2021-07-27

**Authors:** Concetta Ragone, Serena Meola, Pasqualina C. Fiorillo, Roberta Penta, Laura Auriemma, Maria Lina Tornesello, Leonardo Miscio, Ernesta Cavalcanti, Gerardo Botti, Franco M. Buonaguro, Attilio Bianchi, Luigi Buonaguro, Maria Tagliamonte

**Affiliations:** ^1^Innovative Immunological Models Unit, Istituto Nazionale per lo Studio e la Cura dei Tumori, “Fondazione Pascale”—IRCCS, Naples, Italy; ^2^Laboratory Medicine Unit, Istituto Nazionale per lo Studio e la Cura dei Tumori, “Fondazione Pascale”—IRCCS, Naples, Italy; ^3^Ba.S.C.O. Unit, Cellular Manipulation and Immunogenetics, Oncology Department, AORN Santobono-Pausilipon, Naples, Italy; ^4^Molecular Biology and Viral Oncogenesis Unit, Istituto Nazionale per lo Studio e la Cura dei Tumori, “Fondazione Pascale”—IRCCS, Naples, Italy; ^5^Medical Direction, Istituto Nazionale per lo Studio e la Cura dei Tumori, “Fondazione Pascale”—IRCCS, Naples, Italy; ^6^Scientific Direction, Istituto Nazionale per lo Studio e la Cura dei Tumori, “Fondazione Pascale”—IRCCS, Naples, Italy; ^7^General Direction, Istituto Nazionale per lo Studio e la Cura dei Tumori, “Fondazione Pascale”—IRCCS, Naples, Italy

**Keywords:** SARS-CoV-2, HLA-DQ antigens, BNT162b2 mRNA COVID-19 vaccine, antibody titer level, HLA-DR antigens

## Abstract

The response to anti-SARS-Cov-2 preventive vaccine shows high interpersonal variability at short and medium term. One of the explanations might be the individual HLA allelic variants. Indeed, B cell response is stimulated and sustained by CD4^+^ T helper cells activated by antigens presented by HLA-class II alleles on antigen-presenting cells (APCs). The impact of the number of antigens binding to HLA class-II alleles on the antibody response to the COVID vaccine has been assessed in a cohort of 56 healthcare workers who received the full schedule of the Pfizer-BioNTech BNT162b2 vaccine. Such vaccine is based on the entire spike protein of the SARS-CoV-2. Ab titers have been evaluated 2 weeks after the first dose as well as 2 weeks and 4 months after the boosting dose. HLA-DRB1 and DBQ1 for each of the vaccinees have been assessed, and strong binders have been predicted. The analysis showed no significant correlation between the short-medium-term Ab titers and the number of strong binders (SB) for each individual. These results indicate that levels of Ab response to the spike glycoprotein is not dependent on HLA class II allele, suggesting an equivalent efficacy at global level of the currently used vaccines. Furthermore, the pattern of persistence in Ab titer does not correlate with specific alleles or with the number of SBs.

## Introduction

Preventive vaccines are designed to induce neutralizing antibodies able to block infection of target cells by inhibiting the interaction between a pathogen’s surface protein and its cognate cellular receptor.

The vaccination strategy has been based for years on live attenuated or inactivated pathogens, providing the whole array of antigens to the immune system in order to elicit the broadest range of protective antibody response. Recent advances in genomics and proteomics provide essential tools to develop innovative preventive and therapeutic vaccine strategies (i.e., recombinant proteins, synthetic peptides, live-replicating viral vectors, DNA, RNA) that are easy to scale up, safe, and flexible for continuous revision (1).

SARS-CoV-2 is a new human coronavirus identified in patients with acute respiratory syndrome in Wuhan, China, in December 2019 (2). Since then, the SARS-CoV-2 infection has become a pandemic, reaching almost every country in all continents with more than 174 million confirmed cases and over 3.7 million deaths globally (https://gisanddata.maps.arcgis.com/apps/dashboards/bda7594740fd40299423467b48e9ecf6; accessed on June 10, 2021). Physical barriers to block interhuman transmission (i.e., masks, social distancing) have been and are currently implemented with a high grade of success, but the negative social and economic impacts are remarkable. Specific antiviral drugs are not available, and repositioning of those developed and approved for other viruses sharing similar molecular targets has been proposed and tested with limited efficacy (3–5). In this respect, a mass vaccination strategy to build a protective immunity “shield” at individual and population level is of a paramount relevance. The technological advancements in vaccinology have allowed in less than a year to develop a range of vaccines, perform clinical trials, and reach regulatory authorization for the SARS-CoV-2 (6).

The response to anti-SARS-Cov-2 preventive vaccine shows high interpersonal variability at short and medium term (7–9). One of the explanations might be the individual HLA allelic variants. Indeed, B cell response is stimulated and sustained by CD4^+^ T helper cells activated by antigens presented by HLA-class II alleles on antigen-presenting cells (APCs) (10, 11). The Pfizer-BioNTech BNT162b2 vaccine is based on the entire spike glycoprotein of the virus to elicit neutralizing antibodies for blocking the interaction between the virus and the ACE-2 cellular receptor (12). Such a strategy is advantageous for a quick turnaround of COVID-19 vaccine development, but it might reduce the number of potential epitopes to be displayed by the antigen-presenting cells (APCs) in the context of the HLA class II and activate CD4^+^ T helper cells. Indeed, these are considered essential to stimulate and sustain B cell response and antibody production. The strong negative drawback would be that, at global level, only individuals with “favorable” HLA alleles would raise optimal and protective Ab titers.

## Materials and Methods

### Assessment of Ab Titers

Roche Elecsys Anti-SARS-CoV-2 S electrochemiluminescence immunoassay (ECLIA) for the *in vitro* quantitative determination of antibodies (including IgG) against spike RBD of SARS-CoV-2 in human serum was performed on Roche Cobas e 601 module. According to the manufacturer, the correlation test between Roche Elecsys Anti-SARS-CoV-2 S units per ml and WHO International Standards for anti-SARS-CoV-2 immunoglobulins showed an excellent correlation (r^2^ = 0.9992, slope = 0.972, intercept = 0.0072), thus allowing to consider specific Roche Elecsys Anti-SARS-CoV-2 S U/ml units equivalent to WHO International Standard BAU/ml (Binding Arbitrary Units per ml). Measuring range spanned from 0.4 to 2,500.0 BAU/ml; values higher than 0.8 BAU/ml were considered positive.

### Characterization of HLA-DRB1 and DBQ1 Alleles

Genomic DNA was extracted from PBMCs, and a minimum of 40 ng of DNA was used for the reaction. The amplification protocol and the analysis of results were in accordance to the manufacturer (PCR-SSOr LABType SSO, One Lambda Inc, kit: RSSOX1A, RSSOX1B, RSSOX1C, RSSOX2B1, RSSO2Q).

### Epitope Prediction Analysis

Peptides (15 aa long) were predicted with the NetMHCIIpan - 4.0 predictive algorithm (https://services.healthtech.dtu.dk/service.php?NetMHCIIpan-4.0). Peptides for the MHC class II HLA-DRB1 and DQB1 alleles identified in the vaccine have been selected with a predicted %Rank <1 (Strong Binders, SB).

### Statistical Analysis

Comparison between data was performed with the unpaired two-sided Student’s t-test and ANOVA, as appropriate. Normally distributed data were represented as mean ± S.E.M. Two-way ANOVA and Bonferroni *post-hoc* analysis were used to examine the significance of differences among groups. All P values were two-tailed and considered significant if less than 0.05.

## Results

### Pattern of Short-Term Antibody Titers

A cohort of 56 healthcare workers has been enrolled at the National Cancer Institute “Pascale” in Naples, ITALY, upon signing an informed consent. All of them underwent the prescribed schedule of the Pfizer-BioNTech BNT162b2 vaccination (prime at Day 0; boost at day 21).

**Figure 1:**
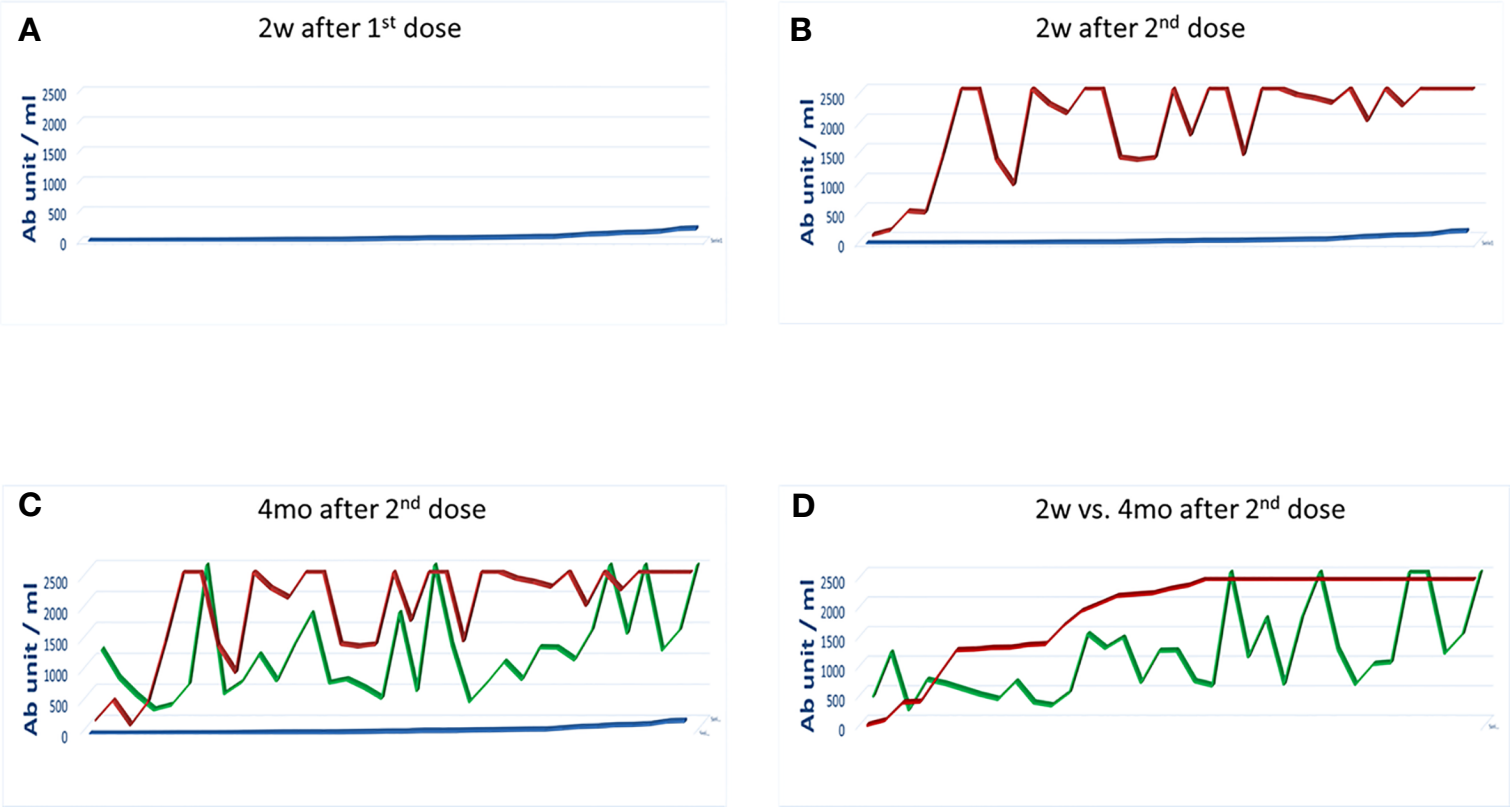
Antibody titers after the 1^st^ and 2^nd^ dose of the Pfizer-BioNTech BNT162b2 vaccine. The Ab titer for each vaccinee is shown 2 weeks after the 1^st^ dose **(A)**; 2 weeks **(B)** and 4 months **(C)** after the 2^nd^ dose. The titers at the two time points after the 2^nd^ dose is shown in **(D)**. In each panel the blue line: 2 weeks after the 1st dose; the red line: 2 weeks after the 2^nd^ dose; the green line: 4 months after the 2^nd^ dose.

Results showed that the response to the 1^st^ dose of vaccine was very low (64.9 BAU/ml on average) with 36 subjects (64.3%) showing an Ab titer <100 BAU/ml, three of which showing 0 BAU/ml, and nine (16%) with an Ab titer between 100 and 550 BAU/ml (**Figure 1A**). Subjects previously recovered from SARS CoV-2 infection showed a titer of 2,500 BAU/ml after the first dose. Therefore, according to Italian rules, they did not receive the second dose of vaccine. Consequently, they were excluded from the subsequent analysis (not shown).

After 2 weeks post-boosting dose, regardless the titer observed after the first vaccine dose, Ab titers showed a 28.8-fold increase (1,872.78 BAU/ml on average). In particular, 99% of the subjects showed an Ab titer >100 BAU/ml, of which 99.5% had a titer >400 BAU/ml and 62.8% had a titer >2,000 BAU/ml. Of note, only one of the vaccinee with 0 BAU/ml after the first dose showed a titer <100 BAU/ml (28 U/ml) also after the second dose (**Figure 1B**).

### Pattern of Medium-Term Antibody Titers

**Figure 2:**
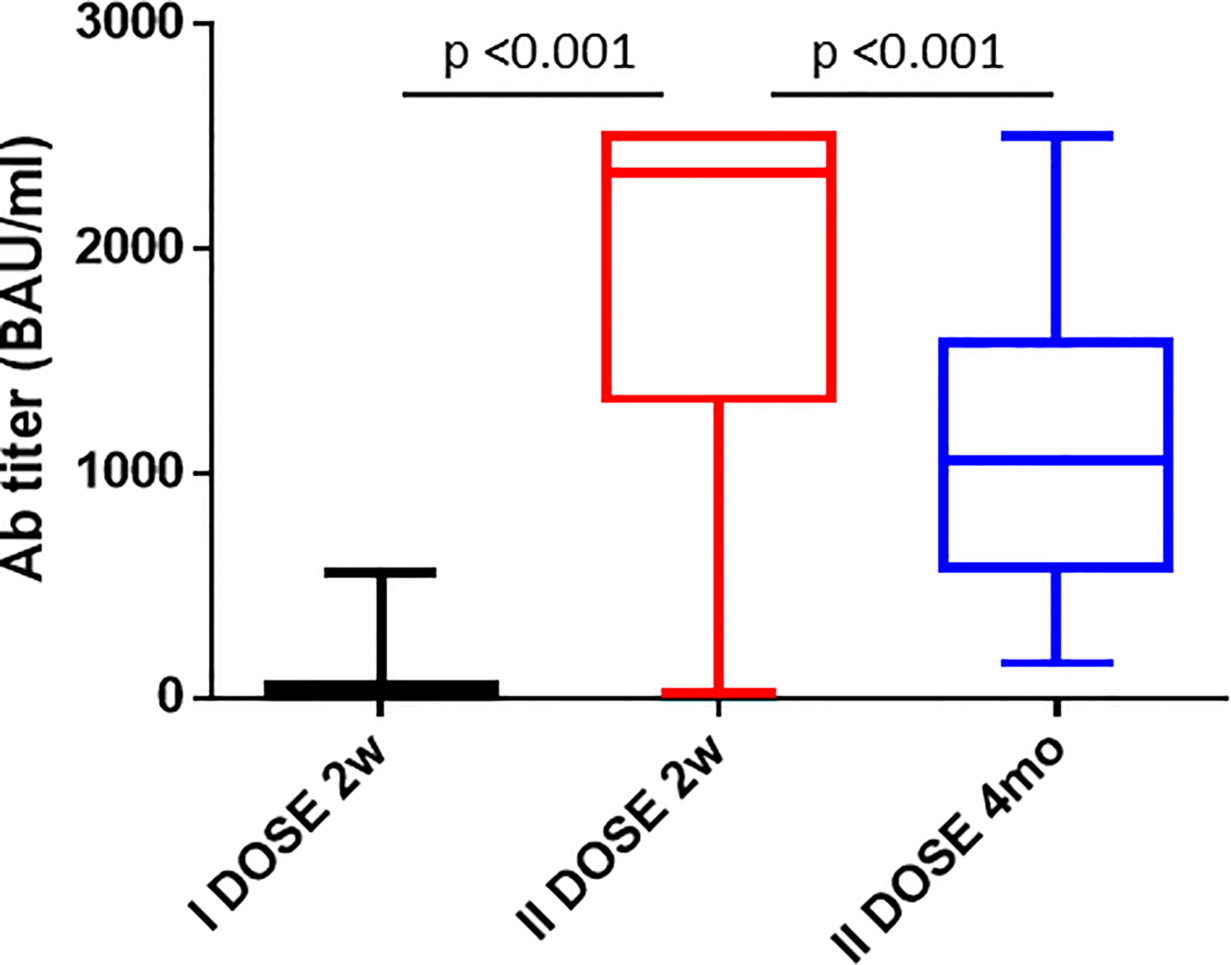
Antibody titers after the 1^st^ and 2^nd^ dose of the Pfizer-BioNTech BNT162b2 vaccine. The cumulative Ab titers in the cohort of enrolled vaccinees is shown 2 weeks after the 1^st^ dose, 2 weeks and 4 months after the 2^nd^ dose.

The serum titer of antibody targeting the RBD was re-evaluated 4 months after the second vaccine dose in 35 subjects of the enrolled cohort. Strikingly, a sharp decline in the Ab titer was observed, and the average value declined from 1,872.78 to 1,063.7 BAU/ml (−56.8%). However, three distinct patterns were observed. An increased Ab titer was observed only in three subjects (8.57%). Subject 1-INT-VAC increased from 27.2 to 388.5 BAU/ml (14.2-fold); subject 4-INT-VAC increased from 423.8 to 670.7 BAU/ml (1.58-fold); subject 5-INT-VAC increased from 106.4 to 1,142 BAU/ml (10.7-fold). The majority of vaccinees (27, 77.14%) showed a sharp decline in Ab titers, with an average decline of 1,138.98 BAU/ml (56.8%), with a percentage of decline ranging from 83.2% (26-INT-VAC) to 25.9% (35-INT-VAC). In such a “declining” subgroup, the Ab titer was 860.19 BAU/ml on average ranging from 1,731 (12-INT-VAC) to 159.7 (20-INT-VAC) BAU/ml. The last subgroup includes five subjects (14.3%) showing a persistence of Ab titers above the threshold of 2,500 BAU/ml (**Figures 1C, D** and **Figure 2**).

### Prediction of HLA Class II Binders

**Figure 3:**
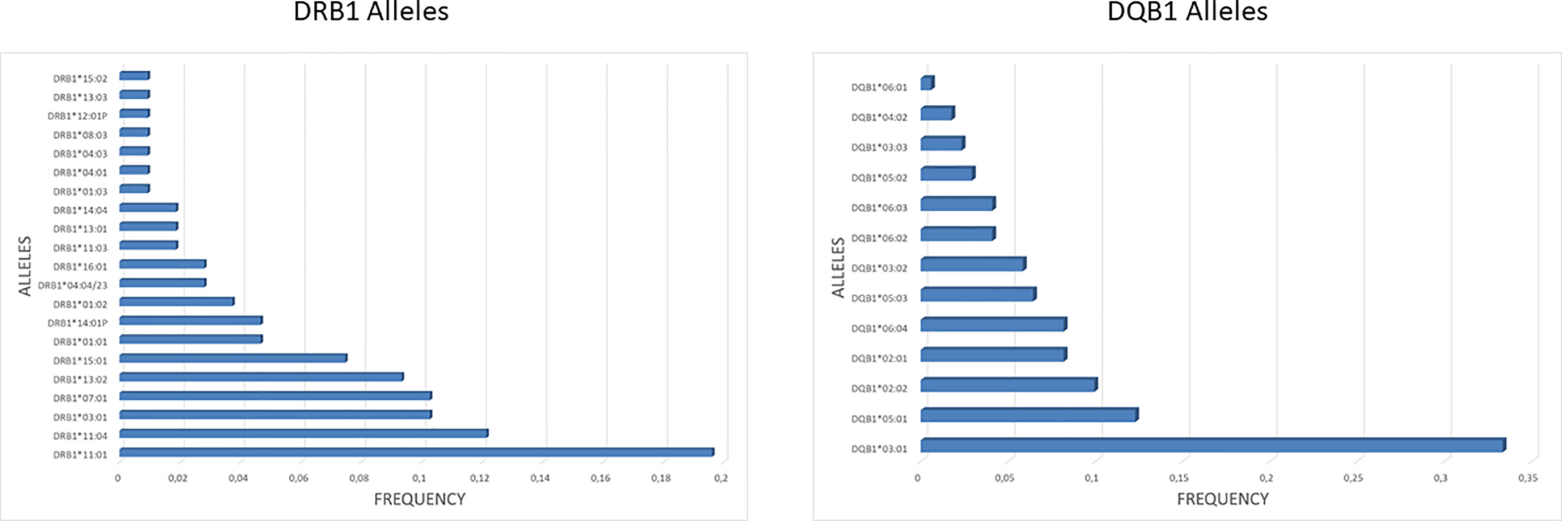
HLA class II frequency in the cohort. The frequency of DRB1 and DQB1 alleles observed in the enrolled subjects is shown. For the DQB1 alleles is shown the cumulative frequency, taking into account all the observed combinations with DQA1 alleles.

In order to identify potential epitopes presented by the HLA class II to CD4^+^ T helper cells, a prediction analysis was performed on the entire sequence of the SARS-CoV-2 spike (UniProtKB - P0DTC2). The HLA class II DRB1 and DQB1 alleles were characterized for each subject enclosed in the enrolled group. The frequency of each allele was significantly different in the group with the DRB1*11:01 and the DQB1*03:01 being the most frequent for the two HLA class II alleles (19.6 and 33.33%, respectively), reflecting the frequency observed in the Italian population (http://www.allelefrequencies.net/) (**Figure 3**).

Each of the alleles returned a great different number of predicted epitopes with high affinity, defined as strong binders (SB) according to the prediction tool. Only those peptides with the predicted highest affinity were taken in consideration (>50% of the threshold value) for the subsequent analysis. Regarding the HLA-DR, the DRB1*15:01 allele returned the highest number of predicted SB (nr. 26) and the DRB1*11:01 allele returned the lowest number of predicted SB (nr. 1). Similarly for the HLA-DQ, the DQA1*05/DQB1*06:04 and DQA1*01/DQB1*06:04 alleles returned the highest number of predicted SB (nr. 24) and the DQA1*01/DQB1*06:01 allele returned the lowest number of predicted SB (nr. 7) (**Tables 1**, **2**). According to such prediction analysis, the subjects included in the cohort showed a highly variable cumulative number of class-II SB ranging from 28 (55-INT-VAC) to 114 (3-INT-VAC), with an average of 70.14 (**Table 3**).

**Table 1:** Number of strong binders for HLA DRB1 alleles identified in the enrolled vaccinees.

DRB1	SB	SB >50% Threshold
DRB1*11:01	6	1
DRB1*13:02	43	22
DRB1*07:01	29	16
DRB1*15:01	42	26
DRB1*03:01	21	13
DRB1*08:03	28	12
DRB1*01:01	17	5
DRB1*13:03	18	5
DRB1*11:04	15	6
DRB1*14:04	28	12
DRB1*01:02	16	3
DRB1*04:04	40	18
DRB1*04:23	40	18
DRB1*12:01	25	9
DRB1*14:01	24	11
DRB1*16:01	28	11
DRB1*11:03	11	5
DRB1*04:03	26	9

**Table 2:** Number of strong binders for HLA DQA1-DQB1 alleles identified in the enrolled vaccinees.

DQA	DQB	SB	SB >50% Threshold
DQA1*01	DQB1*02:01	33	15
DQA1*02	DQB1*02:01	23	8
DQA1*03	DQB1*02:01	20	11
DQA1*05	DQB1*02:01	19	7
DQA1*01	DQB1*02:02	33	15
DQA1*02	DQB1*02:02	23	8
DQA1*05	DQB1*02:02	19	7
DQA1*01	DQB1*03:01	30	17
DQA1*02	DQB1*03:01	25	13
DQA1*03	DQB1*03:01	27	14
DQA1*05	DQB1*03:01	27	16
DQA1*01	DQB1*03:02	35	17
DQA1*02	DQB1*03:02	26	16
DQA1*03	DQB1*03:02	29	15
DQA1*05	DQB1*03:02	30	16
DQA1*02	DQB1*03:03	30	18
DQA1*03	DQB1*03:03	33	20
DQA1*05	DQB1*03:03	32	17
DQA1*03	DQB1*04:02	21	7
DQA1*05	DQB1*04:02	28	13
DQA1*01	DQB1*05:01	26	11
DQA1*02	DQB1*05:01	24	12
DQA1*03	DQB1*05:01	31	13
DQA1*05	DQB1*05:01	25	12
DQA1*01	DQB1*05:02	21	10
DQA1*02	DQB1*05:02	25	8
DQA1*05	DQB1*05:02	34	8
DQA1*01	DQB1*05:03	30	11
DQA1*02	DQB1*05:03	21	7
DQA1*03	DQB1*05:03	23	11
DQA1*05	DQB1*05:03	27	7
DQA1*01	DQB1*06:01	11	7
DQA1*01	DQB1*06:02	27	16
DQA1*05	DQB1*06:02	41	22
DQA1*01	DQB1*06:03	33	18
DQA1*02	DQB1*06:03	34	15
DQA1*05	DQB1*06:03	37	15
DQA1*01	DQB1*06:04	36	24
DQA1*05	DQB1*06:04	38	20

**Table 3:** HLA DR and DQ alleles identified in the enrolled vaccinees.

Code	DRB1	SB	DRB1	SB	DQB1	SB	DQB1	SB	DQB1	SB	DQB1	SB	TOT DRB1	TOT DQB1	TOT SB
3-INT-VAC	DRB1*13:02	22	DRB1*15:01	26	DQA1*01/DQB1*03:01	17	DQA1*05/DQB1*03:01	16	DQA1*01/DQB1*06:02	16	DQA1*05/DQB1*06:02	22	48	71	**119**
13-INT-VAC	DRB1*07:01	16	DRB1*15:01	26	DQA1*01/DQB1*03:01	17	DQA1*05/DQB1*03:01	16	DQA1*01/DQB1*06:03	18	DQA1*05/DQB1*06:03	15	42	66	**108**
10-INT-VAC	DRB1*11:04	6	DRB1*14:04	18	DQA1*01/DQB1*03:01	17	DQA1*05/DQB1*03:01	16	DQA1*05/DQB1*06:04	20	DQA1*01/DQB1*06:04	24	24	77	**101**
1-INT-VAC	DRB1*11:01	1	DRB1*13:02	22	DQA1*01/DQB1*03:01	17	DQA1*05/DQB1*03:01	16	DQA1*01/DQB1*06:04	24	DQA1*05/DQB1*06:04	20	23	77	**100**
7-INT-VAC	DRB1*13:02	22	DRB1*11:01	1	DQA1*01/DQB1*03:01	17	DQA1*05/DQB1*03:01	16	DQA1*05/DQB1*06:04	20	DQA1*01/DQB1*06:04	24	23	77	**100**
4-INT-VAC	DRB1*07:01	16	DRB1*13:01	5	DQA1*01/DQB1*03:01	17	DQA1*05/DQB1*03:01	16	DQA1*01/DQB1*06:04	24	DQA1*05/DQB1*06:04	20	21	77	**98**
29-INT-VAC	DRB1*03:01	13	DRB1*04:04/23	18	DQA1*02/DQB1*03:01	13	DQA1*05/DQB1*03:01	16	DQA1*02/DQB1*03:03	18	DQA1*05/DQB1*03:03	17	31	64	**95**
2-INT-VAC	DRB1*07:01	16	DRB1*11:01	1	DQA1*01/DQB1*03:01	17	DQA1*05/DQB1*03:01	16	DQA1*01/DQB1*06:04	24	DQA1*05/DQB1*06:04	20	17	77	**94**
20-INT-VAC	DRB1*03:01	13	DRB1*12:01P	9	DQA1*01/DQB1*03:01	17	DQA1*05/DQB1*03:01	16	DQA1*01/DQB1*06:02	16	DQA1*05/DQB1*06:02	22	22	71	**93**
23-INT-VAC	DRB1*13:02	22	DRB1*14:01P	11	DQA1*01/DQB1*02:01	15	DQA1*05/DQB1*02:01	7	DQA1*01/DQB1*06:02	16	DQA1*05/DQB1*06:02	22	33	60	**93**
24-INT-VAC	DRB1*13:03	5	DRB1*15:01	26	DQA1*03/DQB1*03:01	14	DQA1*05/DQB1*03:01	16	DQA1*03/DQB1*03:02	15	DQA1*05/DQB1*03:02	16	31	61	**92**
9-INT-VAC	DRB1*13:02	22	DRB1*11:01	1	DQA1*01/DQB1*02:01	15	DQA1*05/DQB1*02:01	7	DQA1*01/DQB1*06:04	24	DQA1*05/DQB1*06:04	20	23	66	**89**
16-INT-VAC	DRB1*11:04	6	DRB1*13:02	22	DQA1*01/DQB1*06:03	18	DQA1*02/DQB1*06:03	15	DQA1*01/DQB1*02:02	15	DQA1*02/DQB1*02:02	8	28	56	**84**
5-INT-VAC	DRB1*03:01	13	DRB1*11:01	1	DQA1*02/DQB1*03:02	16	DQA1*03/DQB1*03:02	15	DQA1*02//DQB1*03:03	18	DQA1*03/DQB1*03:03	20	14	69	**83**
12-INT-VAC	DRB1*07:01	16	DRB1*11:01	1	DQA1*01/DQB1*03:01	17	DQA1*05/DQB1*03:01	16	DQA1*01/DQB1*06:03	18	DQA1*05/DQB1*06:03	15	17	66	**83**
26-INT-VAC	DRB1*11:04	6	DRB1*15:01	26	DQA1*01/DQB1*03:01	17	DQA1*05/DQB1*03:01	16	DQA1*01/DQB1*05:03	11	DQA1*05/DQB1*05:03	7	32	51	**83**
32-INT-VAC	DRB1*07:01	16	DRB1*16:01	11	DQA1*01/DQB1*03:01	17	DQA1*05/DQB1*03:01	16	DQA1*01/DQB1*05:01	11	DQA1*05/DQB1*05:01	12	27	56	**83**
17-INT-VAC	DRB1*04:04/23	18	DRB1*11:01	1	DQA1*01/DQB1*03:01	17	DQA1*05/DQB1*03:01	16	DQA1*01/DQB1*05:01	11	DQA1*05/DQB1*05:01	12	19	56	**75**
21-INT-VAC	DRB1*01:03	8	DRB1*04:04/23	18	DQA1*03/DQB1*02:01	11	DQA1*05/DQB1*02:01	7	DQA1*03/DQB1*03:02	15	DQA1*05/DQB1*03:02	16	26	49	**75**
49-INT-VAC	DRB1*03:01	13	DRB1*15:01	26	DQA1*01/DQB1*05:01	11	DQA1*03/DQB1*05:01	13	DQA1*02/DQB1*05:01	12	—	–	39	36	**75**
15-INT-VAC	DRB1*11:04	6	DRB1*13:02	22	DQA1*01/DQB1*02:02	15	DQA1*02/DQB1*02:02	8	DQA1*01/DQB1*05:03	11	DQA1*02/DQB1*05:03	7	28	41	**69**
25-INT-VAC	DRB1*11:01	1	DRB1*13:02	22	DQA1*02/DQB1*02:02	8	DQA1*01/DQB1*02:02	15	DQA1*01/DQB1*05:01	11	DQA1*02/DQB1*05:01	12	23	46	**69**
18-INT-VAC	DRB1*07:01	16	DRB1*11:01	1	DQA1*01/DQB1*03:01	17	DQA1*05/DQB1*03:01	16	DQA1*01/DQB1*05:03	11	DQA1*05/DQB1*05:03	7	17	51	**68**
38-INT-VAC	DRB1*01:02	3	DRB1*07:01	16	DQA1*01/DQB1*02:02	15	DQA1*02/DQB1*02:02	8	DQA1*01/DQB1*05:01	11	DQA1*02/DQB1*05:01	12	19	46	**65**
11-INT-VAC	DRB1*01:02	3	DRB1*07:01	16	DQA1*05/DQB1*03:01	16	DQA1*02/DQB1*03:01	13	DQA1*05/DQB1*02:02	7	DQA1*02/DQB1*02:02	8	19	44	**63**
14-INT-VAC	DRB1*01:01	5	DRB1*11:01	1	DQA1*01/DQB1*03:02	17	DQA1*03/DQB1*03:02	15	DQA1*01/DQB1*05:01	11	DQA1*03/DQB1*05:01	13	6	56	**62**
27-INT-VAC	—	–	DRB1*11:04	6	DQA1*01/DQB1*03:01	17	DQA1*05/DQB1*03:01	16	DQA1*01/DQB1*05:01	11	DQA1*05/DQB1*05:01	12	6	56	**62**
44-INT-VAC	DRB1*01:01	5	DRB1*15:01	26	DQA1*05/DQB1*03:01	16	DQA1*06/DQB1*03:01	15	—	–	—	–	31	31	**62**
22-INT-VAC	—	–	DRB1*11:04	6	DQA1*01/DQB1*05:03	11	DQA1*03/DQB1*05:03	11	DQA1*01/DQB1*03:02	17	DQA1*03/DQB1*03:02	15	6	54	**60**
40-INT-VAC	DRB1*14:01P	11	DRB1*15:02	25	DQA1*05/DQB1*02:01	7	DQA1*05/DQB1*03:01	16	—	–	—	–	36	23	**59**
6-INT-VAC	DRB1*08:03	12	DRB1*11:01	1	DQA1*02/DQB1*03:01	13	DQA1*05/DQB1*03:01	16	DQA1*02/DQB1*02:02	8	DQA1*02/DQB1*02:02	8	13	45	**58**
28-INT-VAC	—	–	DRB1*11:01	1	DQA1*01/DQB1*03:01	17	DQA1*05/DQB1*03:01	16	DQA1*01/DQB1*05:01	11	DQA1*05/DQB1*05:01	12	1	56	**57**
33-INT-VAC	DRB1*07:01	16	DRB1*14:04	18	DQA1*05/DQB1*02:01	7	DQA1*05/DQB1*03:01	16	—	–	—	–	34	23	**57**
43-INT-VAC	DRB1*04:03	9	DRB1*14:01P	11	DQA1*01/DQB1*03:01	17	DQA1*01/DQB1*06:03	18	—	–	—	–	20	35	**55**
30-INT-VAC	DRB1*01:02	3	DRB1*11:01	1	DQA1*03/DQB1*03:01	14	DQA1*05/DQB1*03:01	16	DQA1*05/DQB1*04:02	13	DQA1*03/DQB1*04:02	7	4	50	**54**
39-INT-VAC	DRB1*03:01	13	DRB1*16:01	11	DQA1*05/DQB1*03:01	16	DQA1*05/DQB1*04:02	13	—	–	—	–	24	29	**53**
8-INT-VAC	DRB1*01:01	5	DRB1*11:03	5	DQA1*01/DQB1*02:02	15	DQA1*02/DQB1*02:02	8	DQA1*01/DQB1*05:02	10	DQA1*02/DQB1*05:02	8	10	41	**51**
34-INT-VAC	—	–	DRB1*07:01	16	DQA1*01/DQB1*05:03	11	—	–	DQA1*01/DQB1*06:04	24	—	–	16	35	**51**
42-INT-VAC	DRB1*03:01	13	DRB1*13:02	22	DQA1*05/DQB1*03:01	16	—	–	—	–	—	–	35	16	**51**
48-INT-VAC	DRB1*01:01	5	DRB1*16:01	11	DQA1*01/DQB1*05:03	11	DQA1*01/DQB1*06:04	24	—	–	—	–	16	35	**51**
51-INT-VAC	DRB1*04:01	19	DRB1*07:01	16	DQA1*05/DQB1*03:01	16	—	–	—	–	—	–	35	16	**51**
53-INT-VAC	DRB1*13:02	22	DRB1*14:01P	11	DQA1*05/DQB1*03:01	16	—	–	—	–	—	–	33	16	**49**
37-INT-VAC	DRB1*11:04	6	DRB1*15:01	26	DQA1*05/DQB1*03:01	16	—	–	—	–	—	–	32	16	**48**
19-INT-VAC	—	–	DRB1*11:01	1	DQA1*01/DQB1*02:02	15	DQA1*02/DQB1*02:02	8	DQA1*01/DQB1*05:01	11	DQA1*02/DQB1*05:01	12	1	46	**47**
35-INT-VAC	DRB1*11:03	5	DRB1*14:01P	11	DQA1*01/DQB1*05:01	11	DQA1*01/DQB1*06:02	16	—	–	—	–	16	27	**43**
50-INT-VAC	DRB1*11:01	1	DRB1*15:01	26	DQA1*05/DQB1*03:01	16	—	–	—	–	—	–	27	16	**43**
41-INT-VAC	DRB1*01:01	5	DRB1*11:04	6	DQA1*02/DQB1*02:01	8	DQA1*05/DQB1*02:01	7	DQA1*02/DQB1*05:02	8	DQA1*05/DQB1*05:02	8	11	31	**42**
46-INT-VAC	DRB1*03:01	13	DRB1*11:01	1	DQA1*05/DQB1*02:01	7	DQA1*05/DQB1*03:01	16	—	–	—	–	14	23	**37**
54-INT-VAC	DRB1*03:01	13	DRB1*11:04	6	DQA1*01/DQB1*05:03	11	DQA1*01/DQB1*06:01	7	—	–	—	–	19	18	**37**
56-INT-VAC	DRB1*03:01	13	DRB1*11:04	6	DQA1*05/DQB1*03:01	16	—	–	—	–	—	–	19	16	**35**
31-INT-VAC	DRB1*01:02	3	DRB1*13:01	5	DQA1*05/DQB1*02:01	7	DQA1*05/DQB1*03:01	16	—	–	—	–	8	23	**31**
36-INT-VAC	DRB1*11:01	1	DRB1*11:04	6	DQA1*05/DQB1*02:01	7	DQA1*05/DQB1*03:01	16	—	–	—	–	7	23	**30**
45-INT-VAC	DRB1*03:01	13	DRB1*11:01	1	DQA1*05/DQB1*03:01	16	—	–	—	–	—	–	14	16	**30**
47-INT-VAC	DRB1*11:01	1	DRB1*11:04	6	DQA1*05/DQB1*03:01	16	DQA1*05/DQB1*02:01	7	—	–	—	–	7	23	**30**
55-INT-VAC	DRB1*11:01	1	DRB1*11:04	6	DQA1*01/DQB1*05:01	11	DQA1*05/DQB1*05:02	8	—	–	—	–	7	19	**26**
52-INT-VAC	DRB1*03:01	13	DRB1*11:01	1	DQA1*02/DQB1*02:02	8	—	–	—	–	—	–	14	8	**22**

Number of predicted strong binders (SB) are indicated for each vaccinee.

### Correlation Between Ab Titer and HLA Class II Strong Binders

**Figure 4:**
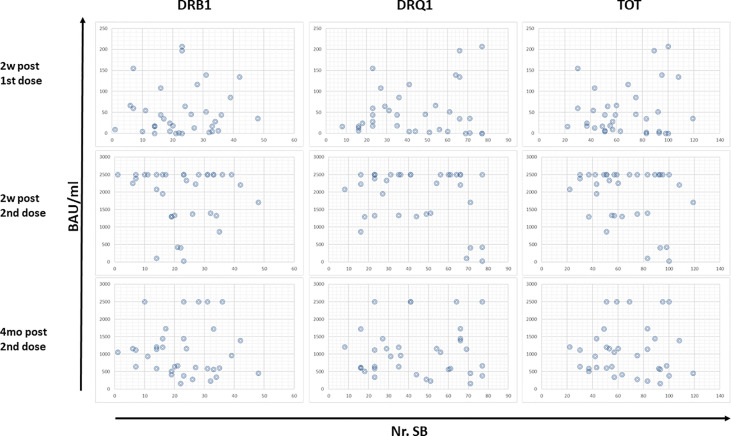
Correlation between Ab titers and predicted SB. The Ab titers at the different timepoints have been correlated with the number of SB predicted for HLA-DR and DQ for each vaccinee. Each circle represents a subject.

For each subject, the Ab titers elicited by the first (2 weeks after) and the second dose (2 weeks and 4 months after) of the vaccine were correlated with the number of SB predicted for the HLA DR and DQ, independently or cumulatively. The results showed that a correlation between number of SB and Ab titers cannot be clearly identified. Indeed, considering the subjects at the extremes (i.e., with the lowest or the highest cumulative number of SB), no difference in Ab titers could be observed at any given timepoint. This is true considering either the HLA-DR, the HLA-DQ, or the sum of both (**Figure 4**).

**Figure 5:**
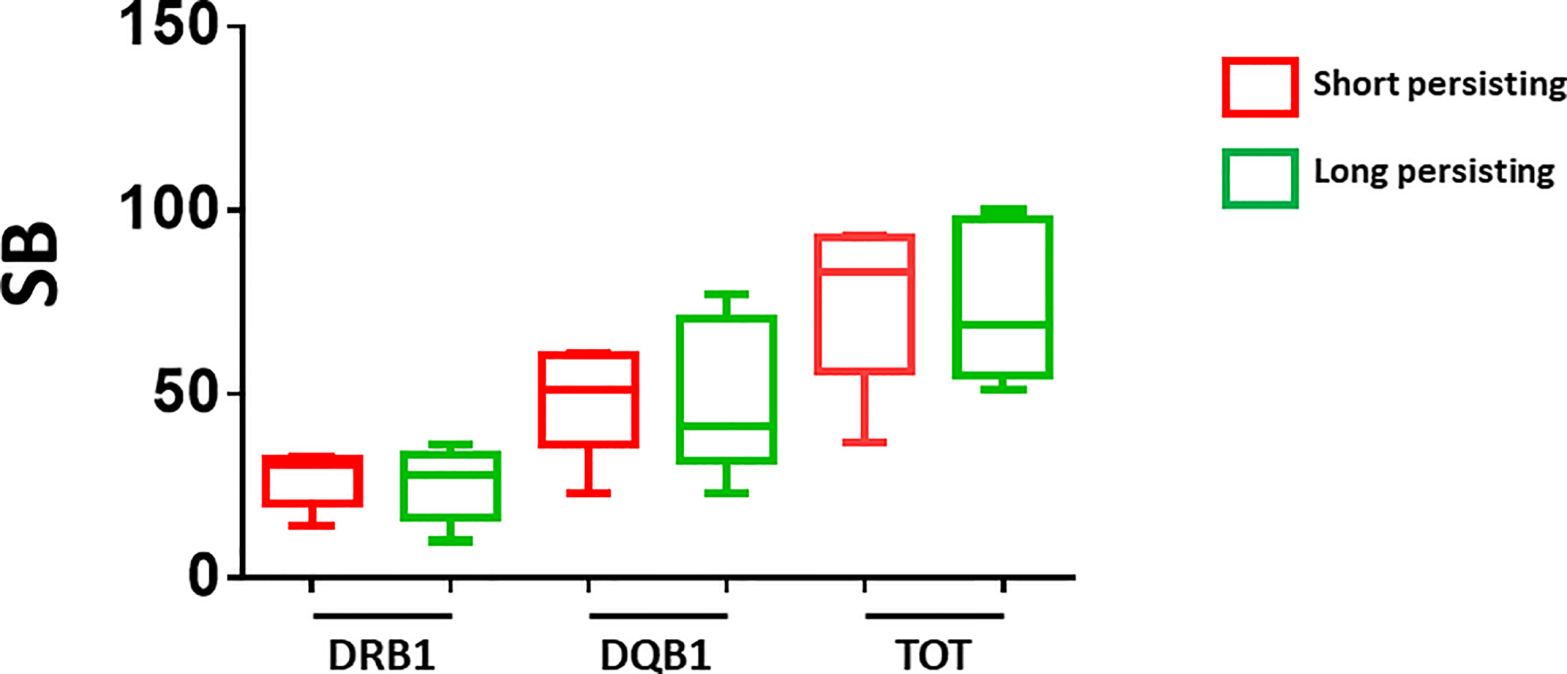
Number of predicted SB in short- and long-persisting vaccinees. The number of strong binders (SB) are indicated for the vaccinees who showed Ab titers persisting at maximum (2,500 BAU/ml) after 4 months (long persisting) and those who showed Ab titers rapidly declining from the maximum after 4 months (short persisting).

Subsequently, we evaluated the alleles and number of SBs in the two subgroups at the extremes of behavior: subjects whose Ab titers persisted at maximum (2,500 BAU/ml) after 4 months (defined as Long Persisting, LP), and subjects whose Ab titers rapidly fell from the maximum after 4 months (75% decline in Ab titer) (defined as Short Persisting, SP). Also in this case, there was no correlation between a specific pattern of DRB1 or DQB1 alleles and the antibody persistence in the LP and SP subgroups (**Table 4**). Consequently, no correlation could be identified with the number of SBs (**Figure 5**).

**Table 4:** HLA DR and DQ alleles identified in the short and long persisting vaccinees.

	Code	Reduction Ab titer (%)	DRB1		DQB1			
**Short persisting**	26-INT-VAC	0,83	DRB1*11:04	DRB1*15:01	DQA1*01/DQB1*03:01	DQA1*05/DQB1*03:01	DQA1*01/DQB1*05:03	DQA1*05/DQB1*05:03
	21-INT-VAC	0,79	DRB1*01:03	DRB1*04:04/23	DQA1*03/DQB1*02:01	DQA1*05/DQB1*02:01	DQA1*03/DQB1*03:02	DQA1*05/DQB1*03:02
	23-INT-VAC	0,77	DRB1*13:02	DRB1*14:01P	DQA1*01/DQB1*02:01	DQA1*05/DQB1*02:01	DQA1*01/DQB1*06:02	DQA1*05/DQB1*06:02
	46-INT-VAC	0,76	DRB1*03:01	DRB1*11:01	DQA1*05/DQB1*02:01	DQA1*05/DQB1*03:01		
	24-INT-VAC	0,76	DRB1*13:03	DRB1*15:01	DQA1*03/DQB1*03:01	DQA1*05/DQB1*03:01	DQA1*03/DQB1*03:02	DQA1*05/DQB1*03:02
**Long persisting**	8-INT-VAC	0,00	DRB1*01:01	DRB1*11:03	DQA1*01/DQB1*02:02	DQA1*02/DQB1*02:02	DQA1*01/DQB1*05:02	DQA1*02/DQB1*05:02
	40-INT-VAC	0,00	DRB1*14:01P	DRB1*15:02	DQA1*05/DQB1*02:01	DQA1*05/DQB1*03:01		
	15-INT-VAC	0,00	DRB1*11:04	DRB1*13:02	DQA1*01/DQB1*02:02	DQA1*02/DQB1*02:02	DQA1*01/DQB1*05:03	DQA1*02/DQB1*05:03
	29-INT-VAC	0,00	DRB1*03:01	DRB1*04:04/23	DQA1*02/DQB1*03:01	DQA1*05/DQB1*03:01	DQA1*02/DQB1*03:03	DQA1*05/DQB1*03:03
	7-INT-VAC	0,00	DRB1*13:02	DRB1*11:01	DQA1*01/DQB1*03:01	DQA1*05/DQB1*03:01	DQA1*05/DQB1*06:04	DQA1*01/DQB1*06:04

## Discussion

The results in a cohort of healthcare workers immunized with the full schedule of the Pfizer-BioNTech BNT162b2 vaccine show a broad range of Ab titers with the majority (62.8%) reaching the >2,000 BAU/ml 2 weeks after the boosting dose. However, only 14.3% of them showed a persistently high Ab titer >2,000 BAU/ml 4 months after the boosting dose. No correlation has been observed between the Ab titers and the number of predicted SB considering either the HLA-DR, the HLA-DQ, or the total. This is true also when comparing subjects whose Ab titers persisted at maximum (2,500 BAU/ml) after 4 months (defined as Long Persisting, LP) and subjects whose Ab titers rapidly fell from the maximum after 4 months (75% decline in Ab titer) (defined as Short Persisting, SP).

In conclusion, the data reported in the present study suggest that the short- and medium-term Ab response to the COVID vaccine is not correlated with the HLA class II allele, suggesting that this is not a parameter of efficacy at global level. This would imply that the present formulation is equally effective everywhere in the World, and the different Ab titers are linked to individual characteristics not related to the HLA background. The impact on the long-term persistence (12 months) of Ab titers needs to be evaluated in the coming period.

## Data Availability Statement

The raw data supporting the conclusions of this article will be made available by the authors, without undue reservation.

## Ethics Statement

The studies involving human participants were reviewed and approved by Institutional Ethical Committee of the Istituto Nazionale Tumori “Pascale”—IRCCS NAPOLI. The patients/participants provided their written informed consent to participate in this study.

## Author Contributions

CR performed the peptide prediction analyses. SM, PF, and EC performed and analyzed the Ab titer evaluation. RP and LA performed the HLA assessment. LM, GB, and AB contributed with productive discussion. MLT and FB contributed to data analysis. MT and LB designed the study, supervised the analysis, and drafted the manuscript. All authors contributed to the article and approved the submitted version.

## Funding

The study was funded by the Campania Region COVID funds.

## Conflict of Interest

The authors declare that the research was conducted in the absence of any commercial or financial relationships that could be construed as a potential conflict of interest.

## Publisher’s Note

All claims expressed in this article are solely those of the authors and do not necessarily represent those of their affiliated organizations, or those of the publisher, the editors and the reviewers. Any product that may be evaluated in this article, or claim that may be made by its manufacturer, is not guaranteed or endorsed by the publisher.
